# Considerations on risk factors correlated to the occurrence of gastric stump cancer

**Published:** 2016

**Authors:** DN Păduraru, A Nica, D Ion, M Handaric, O Andronic

**Affiliations:** * III rd Department of General Surgery, University Emergency Hospital Bucharest, Romania; ** Department of Anesthesiology, University Emergency Hospital Bucharest, Romania; *** ”Carol Davila” University of Medicine and Pharmacy, Bucharest, Romania; **** ”Gr. T. Popa” University of Medicine and Pharmacy, Iași, Romania

**Keywords:** gastric stump cancer, gastric ulcer, duodenal ulcer, gastrectomy

## Abstract

Gastric stump cancer (GSC) is the malignant tumor that develops in the gastric remnant after partial gastrectomy was performed both for benign and malignant lesions. This paper presents the results of the case studies from the scientific literature, which focused on GSC, and has been published in the last 10 years. The search was performed with the help of the specific tools offered by the international databases. The subject was approached because of the constant rising incidence of GSC in the past few years, now reaching values between 1% and 7%. The outcome report is consistent and similar to the period that ended approximately 25 years ago, when general surgeons dedicated a significant part of their activity to treating gastric ulcer. Statistics revealed that the main risk factors are the following: the type of reconstruction after distal gastrectomy (Billroth I or Billroth II), the presence of duodenogastric reflux, the time between gastric resections, and the moment of diagnosis of gastric stump cancer, the initial pathology for which partial gastrectomy was performed, gender, age, helicobacter pylori infection, Epstein Barr virus infection and the presence of vagotomy.

All the authors have significantly contributed to the article and have been involved in the writing of the manuscript in draft and any revision stages, and have read and approved the final version.

## Introduction

Gastric stump cancer (GSC) is the malignant tumor developed in the remnant stomach after the subtotal gastrectomy performed both for benign lesions and for neoplastic disease [**1**-**3**].

The concept of GSC was originally described in the 1920s, and, at that time, it was defined as a carcinoma occurring in the gastric remnant at least 5 years post-surgery for peptic ulcer disease [**4**-**5**]. GSC that developed after distal gastrectomy for cancer was not included in this category. The latter was considered a relapse, its pathogenic mechanisms, histology, and treatment being different. Arbitrarily, some authors defined CBG as a type of metachronous cancer, which occurred during 5 to 10 years after the surgical treatment of primary gastric cancer [**6**].

At present, the concept of CSC was extended and includes both the case of cancer, which occurred after a performed surgery for an initial benign disease and the relapse after the surgical resection of gastric cancer [**7**-**9**]. Accordingly, CBG is described as an adenocarcinoma of the gastric remnant that occurs after 10 years following Billroth I or Billroth II procedure for a benign or malignant disease [**10**].

## Material and method

This paper presents the results of the case studies which focused on the GSC found in the scientific literature and have been published in the last 10 years. The search was performed with the help of the specific tools offered by the international databases.

**Epidemiology**

In 1922, Balfore first reported that the most important factor affecting life expectancy after surgery for gastric ulcer is the occurrence of gastric cancer, which accounts for approximately 40% of the total of deaths [**10**]. The rising number of GSC cases and the association with the operated stomach was described in the 1950s [**11**].

Since surgical therapy was still frequently used for the treatment of gastroduodenal ulcer disease in the 1980s-1990s, and the fact that GSC develops within 20-40 years, an increase around 2015 and 2020 it is expected [**12**-**15**].

In the last years, the incidence of GSC has had an exponential trend [**16**,**17**] and today it ranges between 1% and 7% [**18**-**20**], depending on the clinical trial and the geographical area. The lowest incidence was recorded in Sweden, where its value has not been higher than 1% [**21**-**23**] among the patients who underwent partial gastrectomy, whereas the highest values were reported in the western countries, meaning up to 7% [**24**-**26**].

**Risk factors**

In a meta-analysis that was recently published, the risk factors identified in patients with GSC were evaluated, to better classify the patients with higher risks, who should benefit from adequate surveillance. The statistical data showed that the main risk factors are: the type of reconstruction after distal gastrectomy (Billroth I or Billroth II procedure), the presence of duodenogastric reflux, the time interval between gastric resections and the moment of diagnosis of gastric stump cancer, the initial pathology for which partial gastrectomy was performed, gender, age, Helicobacter Pylori infection, Epstein Barr Virus infection and the presence of vagotomy [**27**].

**Type of reconstruction after distal gastrectomy (Billroth I or Billroth II procedure)**

The two types of partial gastrectomies, which are mentioned in the scientific literature, are Billorth I and Billroth II procedures.

Commonly, patients who are submitted to the Billroth II procedure have a higher risk of developing GSC in comparison with those who had the Billroth I procedure. Moreover, up to 80% of the patients who underwent Billroth II operation have severe duodenogastric reflux [**28**].

In the scientific literature, Billroth II reconstruction performed for gastric ulcer is considered a preneoplastic condition [**29**]. Accordingly, patients submitted to surgery using the Billroth II operation are believed to have a 4-fold increased risk of developing GSC than those who were submitted to the Billroth I reconstruction. In the same vein, Lundegarth et al. revealed that a 4-fold increases the developing of GSC after Billroth II procedure in comparison with the Billroth I procedure in a cohort of 6499 patients [**30**-**31**].

The theme regarding the risk caused by the type of surgery performed is extremely up to date. The answer to the question whether the risk of occurrence of gastric stump cancer is related to the type of reconstruction after partial Billroth I or II gastrectomy, is a debatable concern. As a result of the case studies found in the literature review, it may be concluded that [**32**-**34**]:

● the increased risk with the Billroth II procedure has been attributed to continuous bathing of the gastric stump with secondary bile acids, resulting in mucosal inflammation and regeneration

● Billroth II reconstruction is more frequently associated with atrophic changes and an increased S phase cell count in the proliferative zone

● the interval between primary distal gastrectomy and the diagnosis of GSC is significantly longer in patients treated with Billroth I reconstruction than in those treated with Billroth II reconstruction

● GSC tends to arise from the sites of anastomosis in patients treated with Billroth II reconstruction, in contrast to non-anastomotic sites in patients treated with Billroth I reconstruction.

Taking into consideration both the clinicopathological and the molecular biological changes, the Billorth I procedure is considered to be more suitable in some cases than the Billroth II method, at least when preventing the development of GSC. Furthermore, recently Roux-en-Y reconstruction has been adopted to prevent the gastroduodenal reflux. The time for which the remnant gastric mucosa is exposed to bile reflux is shorter and the degree of remnant gastritis is more mild in patients treated with Roux-en-Y reconstruction than in those treated with Billroth I reconstruction. No reports have suggested that the incidence of GSC is lower in patients treated with Rou-en-Y reconstruction than in those treated with Billroth I reconstruction. However, Roux-en-Y is preferred because it reduces the incidence of duodenogastric reflux and remnant gastric mucosal injury due to gastric carcinogenesis [**35**].

Duodenogastric reflux

GSC is mainly caused by gastroduodenal and bile reflux. Reflux of bile juice to remnant stomach is a major factor in the occurrence of GSC [**36**].

Still, the incidence of GSC is correlated with the duodenogastric reflux [**37**,**38**]. Patients subjected to have a distal gastric resection, have a 4-7 fold increased risk of developing GSC, which is frequently attributed to gastroduodenal reflux.

The data in literature referring to the involvement of duodenogastric reflux in the development of GSC point out the following aspects [**36**-**41**]:

● after the removal of the pylorus there is a reflux of duodenal contents and bile acids. Therefore, the gastric stump is in a constant bath of alkaline reflux. Further, lysolecithin and trypsin digest the gastric mucus. All these factors act together to degenerate the mucosal barrier. Last, but not least, chronic atrophic gastritis develops.

● bile acids such as deoxycholic bile acid, show a carcinogenic effect and may contribute to the carcinogenesis in the gastric stump. The presence of bile acids may induce mucosal injury, gastritis, and finally, cancer in the gastric remnant.

● bile reflux is a well known major pathogenic factor in the gastric remnant reflux gastritis; endoscopic findings of mucosal erythema have been reported to be associated with the alkaline reflux

● secondary to the alkaline reflux, the acid secretion is significantly reduced. There is a free colonization of bacteria. These bacteria are able not only to degrade bile acids into carcinogenic forms, but also convert nitrates to nitrites, leading to the formation of GSC

● the duodenogastric reflux is responsible for premalignant lesions in the anastomotic region, leading more frequently to a diffuse GSC histotype

● many experimental studies focused on bile acids, the main component of the duodenal juice in gastric carcinogenesis; and the results showed that the duodenogastric reflux has a carcinogenic effect in the stomach

● a carcinogenetic influence in the stomach was reported from nitrated derivates of glycocholic and taurocholic bile acids in a rat model

● chronic duodenogastric reflux causes different histological changes in the gastric stump, such as intestinal metaplasia, dysplasia and adenoma

● Helicobacter pylori infection and bile reflux seem to have a synergic effect on cell proliferation in the gastric remnant and may explain the increased risk of cancer after a partial gastrectomy

● most of the patients who underwent a partial gastrectomy complain of dyspepsia symptoms caused by bile reflux

There is a consensus about the fact that the duodenogastric reflux, including bile reflux after the Billroth II procedure is the most common factor encountered in relation to the occurrence of stump carcinoma. 

**The time interval between gastric resections and the moment of diagnosis of gastric stump cancer**

The time interval is one of the most important factors for the development of a GSC, because the gastric remnant is constantly under carcinogenic influence [**31**,**35**].

The average latency time between the distal gastrectomy and the diagnosis of GSC ranges between 20 and 27 years. In rare cases, the latency time may take longer than 40 years [**38**]. Most specialists have reported an increase in the risk of developing GSC from the 20th year after partial gastrectomy. Numerous case studies in literature have stated that the occurrence of gastric stump cancer grows proportionally with the length of the period of time since the beginning of the initial gastric resection [**42**,**43**].

The type of initial gastric disease has a significant impact on the latency of GSC, namely [**28**,**29**,**44**]:

● GSC tends to occur within 25 years following the initial benign disease, and during the 15 years post-operation for patients who have gastric cancer;

● other experts reported that after the surgical treatment of benign lesions, gastric resection is considered a risk factor for GSC even at 15 to 20 years after the surgical treatment, especially when Billroth II reconstruction is performed

● the median interval for patients with benign disease is of 30 years, and for patients with primary gastric cancer is of 12 years

● it takes more than 300 months for GSC to rise from the remnant stomach after distal gastrectomy for benign disease, in contrast to approximately 100 months observation following gastrectomy for primary gastric cancer.

**The initial pathology for which partial gastrectomy was performed**

In a clinical trial published by Tanigawa et al. in 2010, the frequency of CBG at every 5 years after the distal gastrectomy was evaluated by taking into account the original benign or cancer disease. This clinical trial emphasized some important aspects related to the subject of our discussion [**45**,**46**]:

● the patients with original cancer disease developed stump carcinoma in a significantly shorter time interval than those with an original benign disease, especially peptic ulcer disease; patients who were diagnosed with cancer and therefore have already been submitted to surgery have had carcinoma-related gastric mucosal changes at the time of the primary surgery

● for patients with original cancer disease, the reported number of GSC was the highest at 10-15 years after surgery, followed by a gradual decrease in incidence.

The gastric resection for peptic ulcer is still being investigated, for example in patients with refractory ulcer disease and recurrent ulcer with gastric outlet obstruction. For the past 80 years, experts have suggested that there has been an increased risk of gastric cancer after gastrectomy for a benign disease, but it should be taken into account that studies of gastric ulcer patients alone would show an excess risk of GSC, irrespective of the follow-up periods, whereas other studies of the duodenal ulcer patients alone would not indicate an excess risk unless the follow-up was of more than 20 years [**46**].

The explanation of this phenomenon is twofold, as it follows:

● patients with gastric ulcer show a certain degree of gastritis and atrophy in the mucosa of the stomach remnant directly after the operation

● this phenomenon can be similarly observed in patients with duodenal ulcer 20 years after partial gastrectomy.

Secondly, there is a very slight possibility that gastric ulcers treated by surgery have been in fact misdiagnosed with gastric cancers, due to incomplete or even absent histological examination.

Duodenal ulcers are associated with diffuse H. Pylori positive gastritis of the gastric antrum. This particular disease does not have a proven risk for cancer, whereas gastric ulcers occur on a variable background of multifocal atrophic gastritis evolution, which is a premalignant disease [**16**].

The authors reported that in the first group (patients following surgery for benign disease), the duodenal reflux after Billroth II reconstruction leads to the development of carcinoma. In the second group (patients with a time interval of more than 10 years after surgery for malignant disease), a genetic factor such as p53 may be related to metachronous multiple carcinogenesis. For patients of the same group, but with a time interval lower than 10 years after surgery for malignant disease, metachronous multiple carcinogenesis may be associated with diffuse intestinal metaplasia in the mucosa [**16**].

For patients initially operated for gastric cancer, it is still not clear when GSC should be considered a recurrence or a new carcinoma arising on the stump. The recurrence after an early gastric cancer varies between 1,4% and 24% and, generally, occurs within 2 years from surgery. After surgery, it has also been observed at up to 5 years, but rarely after that.

**Gender**

The results of a recent meta-analysis showed that women are less likely to develop GSC than men. Male patients who have undergone a previous surgery through a Billroth II procedure are at the greatest risk of developing GSC [**17**].

Male patients are 4 to 9 times more frequently affected by GSC than females. In a study by Ovasaka et al., the male to female ratio was 36:1, the statistical data in this report being highly significant and strongly contended. The findings of this study can be explained as it follows [**47**]:

● presumed to be a major risk factor for GSC, duodenogastric reflux is more likely to affect men 

● there is a higher incidence of peptic disease in men

● there is a higher risk of men to develop gastric cancer 

● there is a higher frequency of developing gastric resections in men

● lifestyle

● smoking and a higher BMI may be risk factors

● in women, estrogen is a protection against GSC

However, there is no final explanation approved on how the aforementioned factors cause a higher predisposition for CBG in men [**18**].

**Age**

It has been observed that the risk of developing GSC in the remnant stomach is higher for patients who underwent surgery at an early age, namely, below 40 years. Moreover, at the time of the diagnosis, patients with GSC have more than 60 years, and the medium age varies between 67 and 71 years [**48**].

**Helicobacter pylori**

Helicobacter pylori (Hp) infection is a well-known major causative factor of carcinogenesis in the stomach. In primary gastric cancer, Hp may induce carcinogenesis due to the CagA protein, which acts as a growth factor on gastric mucosa cells [**49**].

The influence of this infection on the modifications of the gastric mucosa is controversial [**6**,**7**]. It is worth mentioning that while some specialists state that patients with distal gastrectomy and Hp infection are at an increased risk of developing GSC, others have noticed a drastic decrease of this bacterium in the remnant stomach, experiencing severe duodenal reflux [**36**].

The rate of infection in the gastric stump ranges [**23**]:

● from 50% to 68,2% among all patients treated with distal gastrectomy

● from 55,6% to 72,2% among patients treated with Billroth I reconstruction

● from 58,3% to 66,7% among patients treated with Billroth II reconstruction

The role of Hp in the development of GSC seems different from its effects in the pathogenesis of primary gastric cancer. The degree of inflammation improves and the pH level normalizes following the eradication of Hp in the remnant stomach, therefore the treatment with eradication is recommended to prevent the development of GSC. Matsukura et al. reported that the eradication with double or triple therapy is successful in 70% to 90% of the Hp patients who undergo distal gastrectomy. The higher incidence of Hp infection in early cancer than in locally advanced cancer may suggest the association with the pathogenesis of CBG [**49**].

Nevertheless, unlike primary gastric cancer, Hp infection does not seem to be an important risk factor for GSC, because [**50**]:

● duodenogastric reflux leads to a reduction of Hp growth in the gastric stump

● the rate of infection following gastrectomy gradually decreases over time

● in GSC, the rate of infection is rather low and it ranges between 23% and 28%

● the endoscopic remnant gastritis seems to be caused by the bile reflux, not by Hp infection

● the Hp’s survival and growth may be influenced by an alkaline environment

● the colonization of Hp in the remnant stomach is related inversely to the concentration of bile acids. Danesh reported a drop of 50% of Hp infection rates due to the antibacterial action of bile after gastrectomy

● no significant correlations have been reported between the Hp infection and the carcinogenesis in the remnant stomach

**Epstein Barr Virus infection**

The infection with the Epstein Barr Virus is associated with various cancers, including stomach cancer. In the remnant stomach, the rate of infection varies, as it follows [**50**]:

● from 22,2% to 41,2% among all patients treated with distal gastrectomy

● from 0% to 12,5% among patients treated with Billroth I procedure

● from 30,4% to 58,3% among patients treated with Billroth II reconstruction

Therefore, a higher rate of infection with the Epstein Barr Virus has been discovered in patients treated with Billroth II reconstruction.

**Vagotomy**

The surgical denervation during distal gastrectomy is a cause factor for carcinogenesis. After vagotomy, patients are more prone to epithelial proliferation, and due to this phenomenon, the mucosa is more prone to damage, which eventually would lead to a mutation in the DNA, resulting in the cancer of the remnant stomach [**51**].

Cargyll et al. reported a 7-fold increased risk of GSC in duodenal ulcer patients with vagotomy after the 20th year following surgery.

Factors associated with an increased risk of developing GSC also include the complete denervation of the gastric remnant during the primary gastric resection. The following conclusions regarding this issue should be highlighted [**46**]:

● the increased risk could occur due to hypochlorydia induced by vagotomy

● a consequence of vagotomy is the decrease of defensive factors, including blood circulation to the mucosa, secretion of mucus and renewal of mucosa, leading to the development of GSC

● the intramucosal nerve system plays an important role in controlling the humoral factors, which may lose its extrinsic control by a surgical denervation during gastrectomy

● patients who had a surgical removal of the vagus nerve have lost the gastroprotective function of beta-carotene

● unlike cancer patients, patients with peptic ulcer disease, usually have to undergo either a complete or a partial denervation of the vagus during the surgical resection

● within animal model experiments, rats with an intact vagus nerve exhibited a prostacyclin-induced gastric mucosal protection, which was not observed after a surgical vagotomy. 

The gastroduodenal reflux, the denervation of the stomach and a decrease in gastrin are the main pathological factors responsible for the development of the disease. Distal gastrectomy causes atrophy of the gastric remnant mucosa itself by reducing the levels of antrum-produced hormones like gastrin [**51**].

## Conclusions

The presence of risk factors in patients with a limited gastric resection is an additional reason to monitor them. We found ourselves in a period with an extremely higher number of partial gastrectomies for ulcer in clinical practice. As it could be noticed, all the risk factors that have been identified as a result of the analysis of the scientific literature are enclosed in the group which cannot be changed. This is why the only possibility to cut down the unfortunate consequences of the diagnostics of gastric stump neoplasia, would be the screening programs for the early identification of malignization. Therefore an optimal therapeutic management of the latter, would significantly reduce the morbidity and mortality in patients diagnosed with GSC.

All the authors have significantly contributed and have been involved in the writing of the manuscript in draft and any revision stages, and have read and approved the final version.
